# Mechanisms of ligand recognition and activation of melanin-concentrating hormone receptors

**DOI:** 10.1038/s41421-024-00679-8

**Published:** 2024-05-07

**Authors:** Qian He, Qingning Yuan, Hong Shan, Canrong Wu, Yimin Gu, Kai Wu, Wen Hu, Yumu Zhang, Xinheng He, H. Eric Xu, Li-Hua Zhao

**Affiliations:** 1 State Key Laboratory of Drug Research, Center for Structure and Function of Drug Targets, Shanghai Institute of Materia Medica, Chinese Academy of Sciences, Shanghai, China; 2 University of Chinese Academy of Sciences, Beijing, China; 3 College of Pharmacy, Nanjing University of Chinese Medicine, Nanjing, Jiangsu China

**Keywords:** Cryoelectron microscopy, Cell signalling

## Abstract

Melanin-concentrating hormone (MCH) is a cyclic neuropeptide that regulates food intake, energy balance, and other physiological functions by stimulating MCHR1 and MCHR2 receptors, both of which are class A G protein-coupled receptors. MCHR1 predominately couples to inhibitory G protein, G_i/o_, and MCHR2 can only couple to G_q/11_. Here we present cryo-electron microscopy structures of MCH-activated MCHR1 with G_i_ and MCH-activated MCHR2 with G_q_ at the global resolutions of 3.01 Å and 2.40 Å, respectively. These structures reveal that MCH adopts a consistent cysteine-mediated hairpin loop configuration when bound to both receptors. A central arginine from the LGRVY core motif between the two cysteines of MCH penetrates deeply into the transmembrane pocket, triggering receptor activation. Integrated with mutational and functional insights, our findings elucidate the molecular underpinnings of ligand recognition and MCH receptor activation and offer a structural foundation for targeted drug design.

## Introduction

Melanin-concentrating hormone (MCH) is a cyclic neuropeptide of 19 amino acids, predominantly synthesized by neurons in the hypothalamus and the zona incerta of the brain^1–5^. Originally identified in fish due to its role in melanin aggregation within skin melanophores, MCH in mammals orchestrates a myriad of physiological functions^1^. These range from energy homeostasis, appetite regulation, and sleep–wake cycles to mood modulation, stress responses, and reproductive functions^6–14^. Disruptions in MCH signaling pathways have been associated with obesity, psychiatric conditions, and sleep disorders^15–19^. Mice lacking the MCH system display a lean phenotype, diminished appetite, increased mobile activity, and metabolic shift^20–23^. Consequently, the MCH system is increasingly recognized as a promising therapeutic target for conditions such as obesity, depression, and sleep disorders.

**Fig. 1:**
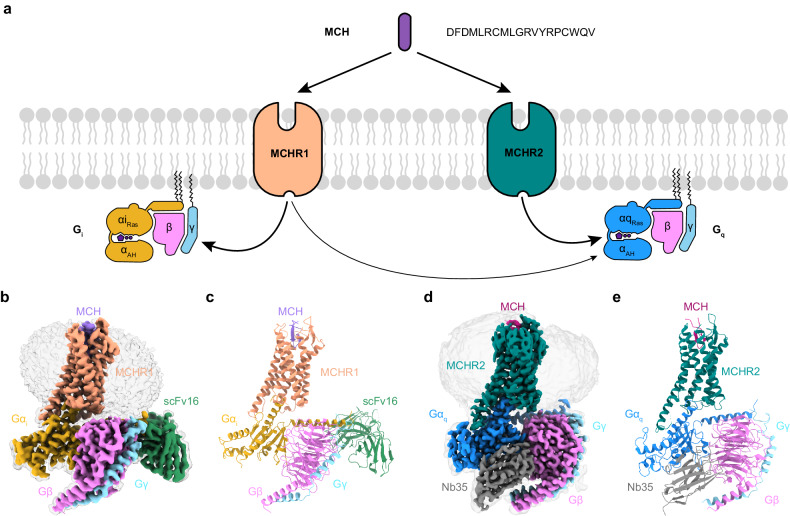
Structural representations of the MCH‒MCHR1‒G_i_ and MCH‒MCHR2‒G_q_ complexes. **a** Depiction of the MCH sequence and a schematic representation illustrating the G-protein coupling of MCHR1 and MCHR2. **b**, **c** Cryo-EM density map (**b**) and the corresponding molecular model (**c**) of the MCH‒MCHR1‒G_i_ complex. **d**, **e** Cryo-EM density map (**d**) and the corresponding molecular model (**e**) of the MCH‒MCHR2‒G_q_ complex.

MCH exerts its effects through two specific G protein-coupled receptors: MCHR1 and MCHR2, both predominantly found in the central nervous system^24^. The binding of MCH to these receptors triggers conformational changes, initiating intracellular signaling cascades via heterotrimeric G proteins. Specifically, MCHR1 predominantly couples with inhibitory G proteins, G_i/o_^24–27^, whereas MCHR2 is exclusively coupled to G_q/11_^24,28^ (Fig. 1a). While MCHR1 antagonists are primarily explored for obesity treatment, they also show promise in treating anxiety and depression^19,29–33^. However, a challenge arises as some antagonists interact with the human *ether-a-go-go-related gene* (hERG) channel, leading to cardiovascular complications^31^. To design drugs with enhanced specificity and minimal off-target effects, a comprehensive understanding of the structural mechanisms of MCH receptor activation is paramount. In this paper, we present cryo-EM structures of MCH-activated MCHR1‒G_i_ and MCHR2‒G_q_ complexes, with resolutions of 3.01 Å and 2.40 Å, respectively. These structures elucidate the molecular details of ligand binding, G protein coupling, and receptor activation, facilitating structure-based drug design targeting MCH receptors.

## Results

### Structure determination and overall structures

In our efforts to stabilize the MCHR1 and MCHR2 complexes for structure determination, we employed multiple strategies to overcome challenges. To enhance cell-surface expression, we implemented an N-terminal truncation of 69 amino acids from MCHR1^5^, following previously reported methods. Additionally, we fused a b562RIL (BRIL) to the N-terminus of both MCHR1 and MCHR2^34^, which has shown to be effective in stabilizing GPCRs. Furthermore, to stabilize the complex, we employed the NanoBiT tethering strategy, attaching the large BiT (LgBiT) to the C-terminus of MCHR1 (residues 70–405) and MCHR2 (residues 1‒331). This arrangement allowed for the formation of a complex with a small BiT (HiBiT) attached to the C-terminus of Gβ^35–37^, as detailed in our methods. Additionally, we appended a dual maltose-binding protein (MBP) tag after the LgBiT to augment receptor expression and facilitate protein purification^38^.

For the assembly of the MCH‒MCHR1‒G_i_ complex, MCHR1, Gα_i_, Gβ, Gγ subunits, and scFv16 were co-expressed in Hi5 insect cells and subsequently incubated with MCH. Accordingly, the MCH‒MCHR2‒G_q_ complex was formed by co-expressing MCHR2, Gα_q_, Gβ, and Gγ subunits in Hi5 insect cells, followed by MCH incubation. The Gα_i_ in the MCHR1 complex harbors dominant-negative mutations (S47N, G203A, A326S, E245A)^39^, which diminish nucleotide-binding affinity and stabilize the Gαβγ heterotrimer complex. The Gα_q_ in the MCHR2 complex is a mini-Gα_q_, which permits the addition of nanobody (Nb35) to bolster the stability of the receptor‒G protein complex. The Gβ subunit in both MCHR1 and MCHR2 complexes contains a C-terminal HiBiT, which interacts with the LgBiT at the C-terminus of receptors, effectively anchoring the G protein complex to the receptor.

The structures of the MCH‒MCHR1‒G_i_ and MCH‒MCHR2‒G_q_ complexes were resolved by cryo-EM to 3.01 and 2.40 Å, respectively (Fig. 1b‒e; Supplementary Figs. S1, S2 and Table S1). The clarity of the cryo-EM density maps allowed for the accurate modeling of most side chains, encompassing MCH, the receptors, the Gαβγ heterotrimer, and both scFv16 and Nb35 (Fig. 1c, e; Supplementary Fig. S3). In terms of receptor composition in the final structure models, MCHR1 spans residues 107–393, and MCHR2 covers residues 31–317. Regarding the ligand, the residues 2‒18 of MCH in the MCH‒MCHR1‒G_i_ complex are well-defined. However, in the MCH‒MCHR2‒G_q_ complex, the N-terminal 4 amino acids of MCH are absent from the EM map.

The overall structures of MCHR1 and MCHR2 in both complexes are highly similar, adopting a canonical seven transmembrane helix domain (TMD), with root mean square deviation (RMSD) values of 1.009 Å over 226 Cα atoms for the receptors and 1.874 Å over 778 Cα atoms for the complete complexes. The ligand, MCH, also adopts a similar γ-shaped structure in both receptor complexes, with the RMSD value of 0.51 Å for the central 10 residues between the two disulfate-bond cysteines (C7 and C16). The binding site of MCH in both receptors is at the orthosteric pocket formed by transmembrane helices TM2, TM3, and TM5‒7, as well as three extracellular loops (ECL1‒3) (Supplementary Tables S2‒S4). The overall binding mode of MCH to MCHR1 and MCHR2 is similar to the binding mode of other cyclic peptides, including somatostatin-14 (SST14) to somatostatin receptor 2 (SSTR2) and arginine vasopressin (AVP) to vasopressin type 2 receptor (V2R).

### MCH recognition by MCHR1

**Fig. 2:**
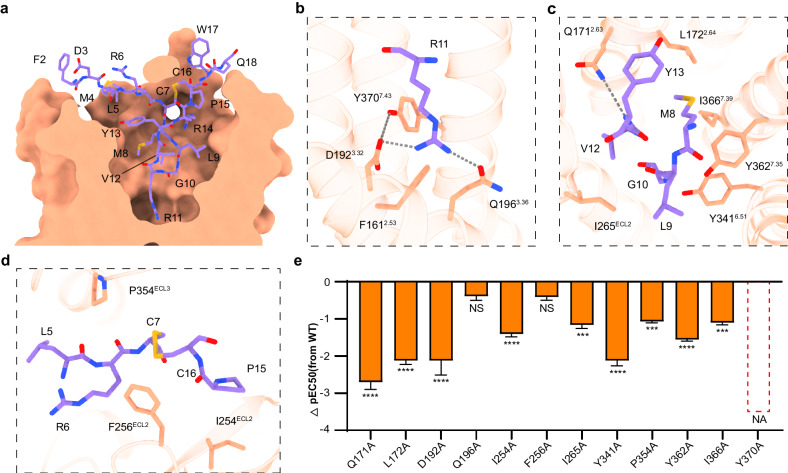
Molecular basis of MCH recognition by MCHR1. **a** Cross-section of the MCH-binding pocket in MCHR1. **b**‒**d** Detailed molecular interactions between MCH (depicted in medium purple) and MCHR1 (shown in light salmon). Hydrogen bonds are presented with gray dashed lines. **e** Functional implications of mutations within the MCHR1 binding pocket, represented as ΔpEC50 values. ΔpEC50 represents the difference in pEC50 values between the WT and the mutants of MCHR1. Data from three independent experiments, each of which was performed in triplicate, are presented as mean ± SEM. Statistical differences were determined by two-sided one-way ANOVA with Tukey’s test compared with WT. NA no activity; NS no significant difference; *P < 0.05; **P < 0.01; ***P < 0.001; ****P < 0.0001.

MCH in the MCH‒MCHR1 complex adopts a γ-shaped configuration, with the central 10 residues forming a cyclic loop with a disulfate bond by cysteine residues at positions 7 and 16. The central loop residues are buried in the TMD pocket, with the central arginine from the conserved LGRVY core motif (residues 9‒13) inserted deeply into the bottom of the pocket. The N-terminal portion of MCH (residues 2‒6) runs toward TM2 and the extracellular loop 1 (ECL1), where the C-terminal portion of MCH (residues 17 and 18) runs towards TM5 and packs along with the extracellular loop 2 (ECL2) (Fig. 2a).

The binding mode of MCH in MCHR1 is stabilized by extensive interactions of MCH with specific residues in the receptor binding pocket, as summarized in Supplementary Table S2. The most prominent interaction is mediated by R11 of MCH, the side chain of which is docked near the bottom of the TMD pocket and forms a salt bridge with D192^3.32^, a hydrogen bond with Q196^3.36^ and cation–π interaction with Y370^7.43^ (Fig. 2b). Additionally, D192^3.32^ forms a hydrogen bond with Y370^7.43^. Surrounding the central arginine are several hydrophobic residues (the LGRVY motif), which form extensive hydrophobic interactions with residues F161^2.53^, L172^2.64^, I265^ECL2^, Y341^6.51^, Y362^7.35^ in the TMD pocket (Fig. 2b, c). Besides, the main chain of Y13 forms a hydrogen bond with Q171^2.63^ (Fig. 2c). Other hydrophobic residues within the cyclic loop (M8 and P15) also make hydrophobic interactions with receptor residues (Y362^7.35^, I366^7.39^, and I254^ECL2^) in the upper part of the TMD pocket (Fig. 2c, d). In addition, the hydrophobic residue of L5 and the hydrophobic portion of R6 contact residues from ECL2 and ECL3, including F256^ECL2^ and P354^ECL3^ (Fig. 2d). Alanine mutation at key pocket residues, led to varied degrees of reduced receptor activation, with the Y370^7.43^ mutation abolishing activity completely, highlighting the importance of these interactions (Fig. 2e; Supplementary Fig. S4 and Table S5).

### MCH recognition by MCHR2

**Fig. 3:**
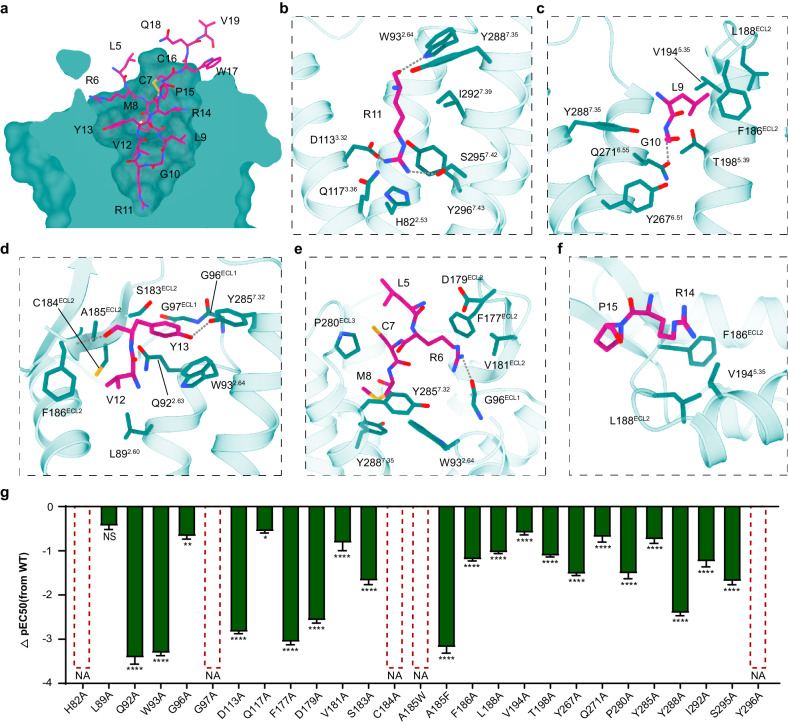
Molecular basis of MCH recognition by MCHR2. **a** Cross-section of the MCH-binding pocket in MCHR2. **b**‒**f** Detailed molecular interactions between MCH (depicted in medium violet red) and MCHR2 (shown in teal). Hydrogen bonds are highlighted with gray dashed lines. **g** Functional data derived from alanine mutations of residues within the MCHR2 binding pocket, represented as ΔpEC50 values. ΔpEC50 represents the difference in pEC50 values between the WT and the mutants of MCHR2. Data are presented as mean ± SEM. from three independent experiments performed in triplicate and analyzed by two-sided one-way ANOVA with Tukey’s test compared with WT. NA no activity; *P < 0.05; **P < 0.01; ***P < 0.001; ****P < 0.0001.

The mode of MCH recognition by MCHR2 closely resembles that by MCHR1, with the MCH displaying a similar binding pose. The central arginine from the conserved LGRVY core motif is also deeply buried into the bottom of the orthosteric pocket of MCHR2 (Fig. 3a). Overall, MCH engages in similar interactions with both MCHR1 and MCHR2, as summarized in Fig. 3b‒f; Supplementary Fig. S5 and Tables S2‒S4. However, MCH makes more extensive and tighter interactions with the residues in MCHR2 than in MCHR1, especially with ECL2 of MCHR2 (Fig. 3c‒f).

In addition to various similar interactions, MCH and MCHR2 engage in additional polar interactions. The central arginine of MCH can form hydrogen bonds with W93^2.64^ and S295^7.42^ of MCHR2 (Fig. 3b). The corresponding positions of MCHR1 are substituted by L172^2.64^ and G369^7.42^, which cannot form hydrogen bonds with R11. Among the other residues of the core motif, the backbone amine group of G10 and the backbone carbonyl group of Y13 can form hydrogen bonds with Q271^6.55^ and the main chain amine group of F186^ECL2^, respectively, and the hydroxyl group of Y13 and Y285^7.32^ can form a hydrogen bond (Fig. 3c, d). The sidechain of R6 forms electrostatic interaction with D179^ECL2^ and a hydrogen bond with the backbone carbonyl of G96^ECL1^ (Fig. 3e). In addition, ECL2 of MCHR2 forms a hydrophobic cap composed of F177^ECL2^, V181^ECL2^, S183^ECL2^, C184^ECL2^, A185^ECL2^, F186^ECL2^, and L188^ECL2^, which makes intensive hydrophobic interactions with MCH residues L5, L9 and V12‒P15 (Fig. 3c‒f).

Consistently, alanine mutations of the aforementioned receptor pocket residues significantly decrease or even abolish receptor activity, highlighting the essential role of these amino acids in the MCH binding to MCHR2 (Fig. 3g; Supplementary Fig. S4 and Table S6). In addition, MCH is closely packed against ECL2 of MCHR2 (Fig. 3c‒f). The A185^ECL2^F mutation results in a decrease in activity by more than 1000-fold, while the A185^ECL2^W mutation completely abolishes activity (Fig. 3g). This suggests that increased steric hindrance of ECL2 may hinder MCH from effectively accessing the ligand-binding pocket.

### Differences in MCH binding between MCHR1 and MCHR2

**Fig. 4:**
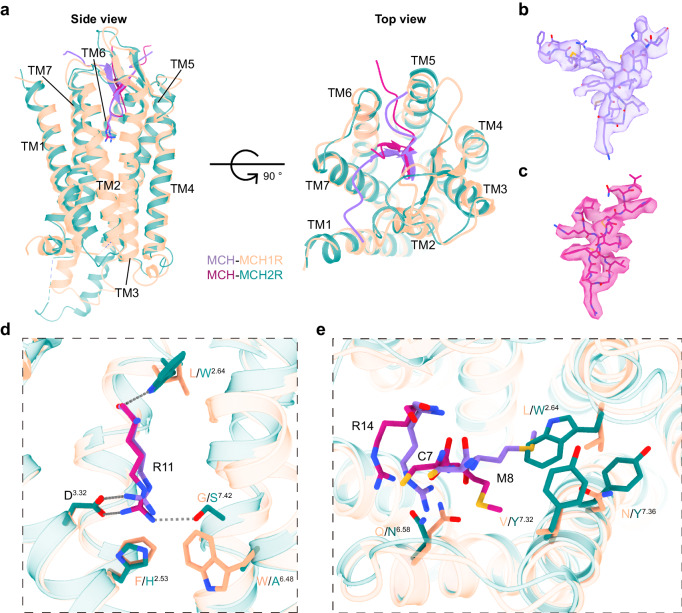
Conformational differences of MCH recognition by MCHR1 and MCHR2. **a** Structural superposition of MCH-bound MCHRs, presented from both side-view (the left) and top view (the right). **b**, **c** Mesh representations of MCH within the binding pockets of MCHR1 and MCHR2. **d**, **e** The differential interactions between MCH and MCHRs, focusing on R11 (**d**) and the C7, M8, and R14 residues of MCH (**e**).

While MCHR1 and MCHR2 share highly similar overall structures and MCH binding modes, subtle differences in their binding pockets confer differences in MCH binding (Fig. 4a‒c). Notably, residues deep within the binding pocket of MCHR2 engage in extensive interactions with the critical R11 of MCH. R11 forms hydrogen bonds with S^7.42^ and W^2.64^ in MCHR2, enabling a unique interaction pattern (Fig. 4d). In contrast, in MCHR1, R11 shifts away from TM6 due to a potential steric clash with W^6.48^ and the absence of a hydrogen bond with G^7.42^ (corresponding to A^6.48^ and S^7.42^ in MCHR2) (Fig. 4d).

Additionally, residues 2.64 and 7.32 confer other differences. MCHR1 has L^2.64^ and V^7.32^, while MCHR2 has W^2.64^ and Y^7.32^. The bulky tryptophan and tyrosine residues introduce steric hindrance that brings C7 and M8 of MCH closer to TM4 and TM5 in MCHR2 (Fig. 4e). This enhances interactions between MCH and ECL2 of MCHR2, which dips slightly towards the binding pocket. Furthermore, R14 of MCH clashes with Q^6.58^ in MCHR1, causing it to shift downwards compared to N^6.58^ in MCHR2 (Fig. 4e). Collectively, these distinctions display unique differences between MCH binding to MCHR1 and MCHR2.

### Activation mechanism of MCHRs

**Fig. 5:**
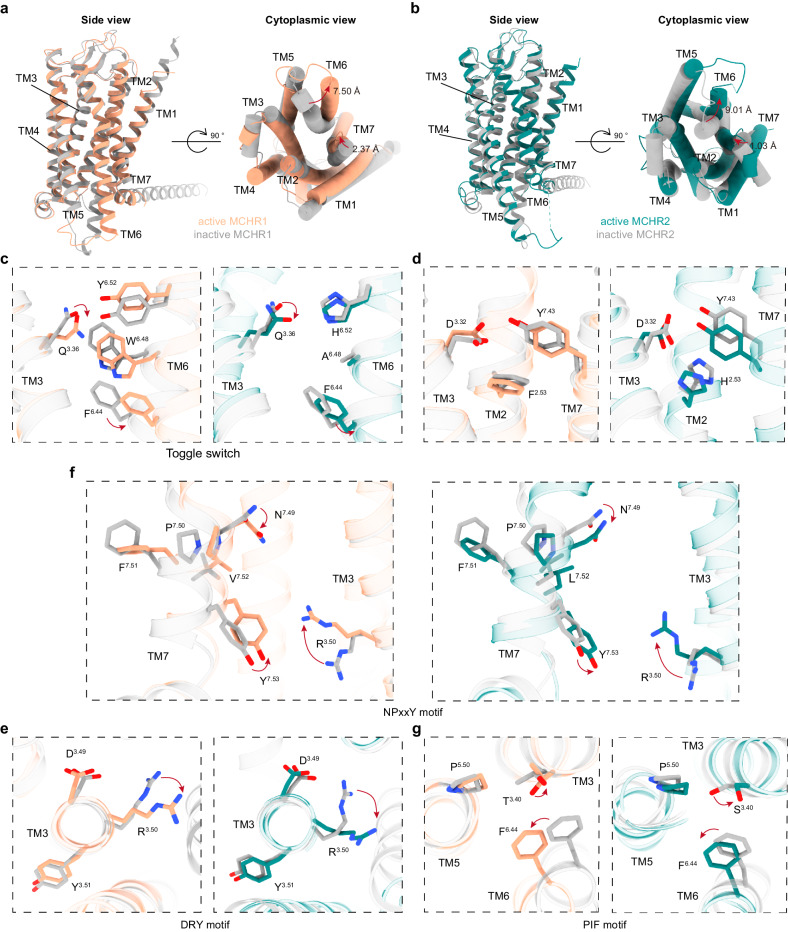
Conformational changes in MCHR1 and MCHR2 upon activation. **a**, **b** Structural superposition between cryo-EM structures of MCH-bound MCHR complexes and the AlphaFold2-predicted inactive models from the GPCR databank is shown from the side view and the cytoplasmic view. **c** Structural alignment highlighting the toggle switch mechanism. **d** Depiction of shifts in H/F^2.53^, D^3.32^, and Y^7.43^ at the base of the binding pocket. **e**‒**g** Illustration of the conserved motifs in MCH-activated MCHRs: DRY motif (**e**); NPxxY motif (**f**); PIF motif (**g**).

To elucidate the activation mechanisms of MCHR1 and MCHR2, we compared their active structures to the AlphaFold2-generated inactive models from GPCR databank (Fig. 5). Structural alignment showed that upon MCH binding and activation, the cytoplasmic ends of TM6 in MCHR1 and MCHR2 moved outward by approximately 7.50 Å and 9.01 Å respectively, as measured at the Cα of residue 6.31 (Fig. 5a, b). This outward movement of TM6 is a hallmark of GPCR activation and enables coupling to downstream G proteins. Additionally, TM7 underwent an inward shift of 2.37 Å in MCHR1 and 1.03 Å in MCHR2 at the Cα of residue 7.53 (Fig. 5a, b). The combination of TM6 and TM7 movements opens a cleft on the intracellular side to accommodate G protein binding.

The activation of MCHR1 and MCHR2 is intricately regulated by MCH binding. This interaction occurs within the orthosteric binding pocket, leading to conformational changes that extend through the helices. However, contrary to the commonly observed shift of the conserved toggle switch W^6.48^ in most class A GPCRs, we observed minimal movement of the tryptophan in MCHR1 and an alanine substitution at this position in MCHR2 (Fig. 5c). Instead, our study reveals a novel mechanism of activation for these receptors. MCH disrupts the polar interaction between Q^3.36^ and H/Y^6.52^, leading to the displacement of these residues, a critical event for receptor activation (Fig. 5c). This was further substantiated by our mutagenesis experiments. In MCHR1, mutations at Q196^3.36^ and Y342^6.52^ increased basal activity by approximately 20%, confirming the significance of disrupting this polar interaction for receptor activation (Supplementary Fig. S4a‒d). In contrast, similar mutations in MCHR2 resulted in distinct outcomes: the H268^6.52^ to F mutation led to receptor inactivity, and the Q117^3.36^A mutation had a minimal impact (Supplementary Fig. S4b‒e). These findings highlight a unique activation mechanism for MCHR2, divergent from that of MCHR1. The differential effects observed upon mutating Q^3.36^ and H/Y^6.52^ between MCHR1 and MCHR2 provide a deeper understanding of the activation mechanisms in these receptors, emphasizing the complexity and diversity of GPCR activation.

Furthermore, R11 of MCH forms critical interactions with D^3.32^ and Y^7.43^ at the base of the pocket. This results in downward shifts of D^3.32^ and Y^7.43^, which in turn induces the upward movement of R^3.50^ in the DRY motif and subsequent downward shift of Y^7.53^ in the NPxxY motif (Fig. 5d‒f). The H/Y^6.52^ displacement also shifts F^6.44^ in the PIF motif (Fig. 5g). Collectively, these rearrangements within key structural motifs facilitate the activated state.

### G-protein coupling of MCHR1 and MCHR2

MCHR1 and MCHR2 exhibit distinct G-protein coupling profiles. Specifically, MCHR1 is capable of associating with a diverse set of G-proteins, namely Gα_i/o_ and Gα_q/11_. Conversely, MCHR2 demonstrates specificity by predominantly coupling to Gα_q/11_. Experimental analyses further elucidated that MCHR1 manifests a heightened efficacy in its coupling to Gα_i_ as opposed to Gα_q_, as depicted in Supplementary Fig. S4c.

**Fig. 6:**
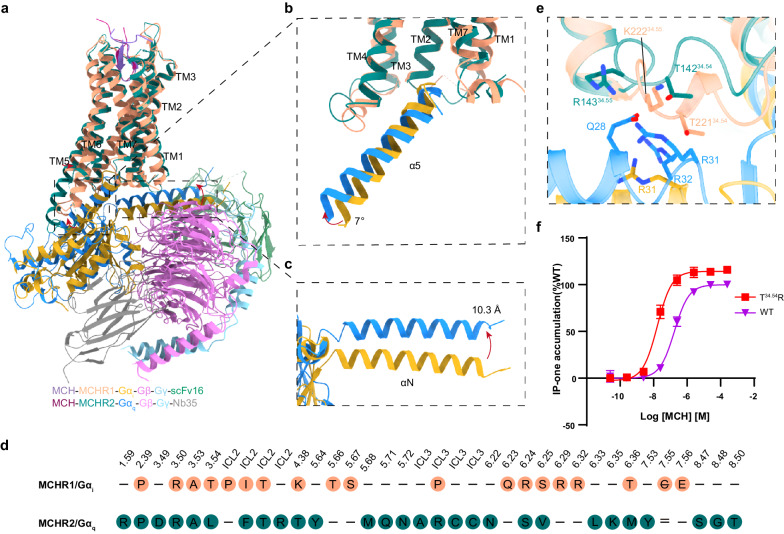
G-protein coupling of MCHR1 and MCHR2. **a** Structural alignment between MCHR1‒G_i_ and MCHR2‒G_q_ complexes. **b**, **c** Comparative analysis of the Gα conformation in the α5 (**b**) and αN (**c**) domains. **d** Key residues in MCHR1 and MCHR2 that interact with the downstream Gα_i_ or Gα_q_ proteins. **e** Detailed interactions between the ICL2 of MCHR1 and MCHR2 with the αN domain of Gα_i_ or Gα_q_. **f** IP-one assay data for wild-type and T221^34.54^R of MCHR1, with data presented as mean values ± SEM from three independent experiments performed in triplicate.

Structural alignments between Gα_i_ and Gα_q_ revealed notable conformational differences (Fig. 6a). The α5 helix of both Gα_i_ and Gα_q_ exhibits a deflection of ~7°, and a discernible spatial separation of ~10.3 Å is observed in their respective αN regions (Fig. 6b, c). Intriguingly, both MCHR1 and MCHR2 engage with Gα_i_ or Gα_q_ through transmembrane domains 2–7 (TM2‒7) and intracellular loops 2 and 3 (ICL2 and ICL3) (Fig. 6d). Additional interactions via TM1 and Helix8 are uniquely observed in MCHR2 (Fig. 6d).

A critical feature of Gα_q_-coupled receptors is the presence of a basic residue, typically arginine or lysine, at positions 34.54 or 34.55 within ICL2^40^. In MCHR1, the threonine residue at position 34.54 (T221) engages in a polar interaction with the αN domain of Gα_q_ (Fig. 6e). Interestingly, when this threonine was mutated to arginine, there was a significant tenfold increase in the coupling efficiency of MCHR1 to Gα_q_, underscoring the importance of this residue in G-protein coupling specificity (Fig. 6f). In contrast, a similar mutation in MCHR2 (T142^34.54^ to R) did not enhance the coupling efficiency to Gα_q_ (Supplementary Fig. S4e). This disparity in functional outcomes between MCHR1 and MCHR2 suggests that the determinants of G-protein coupling specificity extend beyond a single residue alteration. The lack of a similar effect in MCHR2 indicates the complexity of GPCR‒G protein interactions, where a broader network of interactions and structural conformations plays a crucial role. Thus, while T^34.54^ is a pivotal element in modulating G_q_ coupling in MCHR1, its role in MCHR2 appears to be distinct, likely influenced by other structural factors and residues. These findings highlight the intricate and receptor-specific nature of GPCR‒G protein interactions, emphasizing that insights derived from one receptor subtype cannot be directly transferable to another, even within the same receptor family. Our study provides an understanding of G-protein coupling specificities in MCHR1 and MCHR2, revealing the complex interplay of residues and structural elements that govern these interactions.

### Comparison of ligand-activated SSTR2 and V2R

**Fig. 7:**
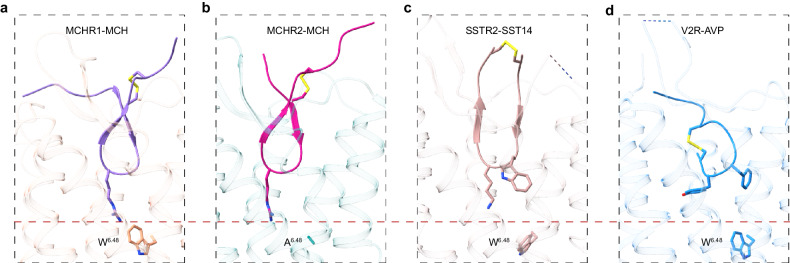
Distinct binding modes of cyclic peptides in receptors. **a** Binding conformation of MCH in MCHR1. **b** Binding conformation of MCH in MCHR2. **c** Binding conformation of SST14 in SSTR2 (PDB: 7Y27). **d** Binding conformation of AVP in V2R (PDB: 7DW9).

MCH, SST14, and AVP are cyclic polypeptides, each stabilized by disulfide bonds. These peptides adopt analogous conformations, with their cyclic segments deeply embedded within their respective receptor binding pockets (SST14 in SSTR2; AVP in V2R; Supplementary Fig. S6). Distinctively, MCH adopts a twisted γ-shaped ring conformation, diverging from the near-planar cyclic amino acid backbones exhibited by SST14 and AVP (Fig. 7).

SST14, characterized by its expansive loop encompassing 12 amino acids, positions its disulfide bond above the receptor pocket. Central residues, W8 and K9, from its FWKT motif, play pivotal roles in receptor engagement (Fig. 7c). In contrast, AVP, with its compact 6-amino acid ring, situates its disulfide bond intrinsically within the receptor pocket (Fig. 7d). Key residues, Y2 and F3, delve into the pocket’s depths, with their side chains docked horizontally into the pocket, which contrasts with the vertical docking of the sidechain of R11 from MCH into both MCHR1 and MCHR2 pocket (Fig. 7a, b and d). The profound penetration of MCH into the receptor pocket surpasses that of both SST14 and AVP.

### Analysis of AlphaFold2 predictions and cryo-EM structures

In our endeavor to comprehensively understand the structural aspects of MCHR1 and MCHR2, we employed AlphaFold2 to predict the structures of the MCHR1‒G_i_ and MCHR2‒G_q_ complexes. This comparison aimed to assess the accuracy and limitations of computational predictions in contrast to experimental cryo-EM data. The AlphaFold2 models exhibited remarkable similarity to our cryo-EM structures (Supplementary Fig. S7a), with RMSDs of 0.988 Å over 229 Cα atoms for MCHR1 and 1.249 Å over 242 Cα atoms for MCHR2. These low RMSDs underscore the impressive accuracy of AlphaFold2 in predicting the overall architecture of these GPCRs.

Despite the overall structural congruence, we identified several key differences between cryo-EM structures and computational predictions. In the AlphaFold2 models, the MCH ligand exhibited a short alpha-helical conformation for residues 2‒7, diverging significantly from the short beta-strand configuration observed in our cryo-EM structures (Supplementary Fig. S7). This distinction critically alters the ligand‒receptor interaction and the shape of the binding pocket. The altered conformation of MCH in the AI models resulted in an expanded binding pocket, with observable outward movements in ECL2, ECL3, TM1, and TM2 (Supplementary Fig. S7b, d). These structural adjustments in response to the ligand’s conformation highlight the sensitivity of receptor architecture to ligand shape. We also noted significant outward movements in the cytoplasmic end of TM6 in the AI models, with a corresponding 2‒3 Å displacement of the G protein heterotrimer (Supplementary Fig. S7a). These deviations suggest differences in the predicted vs actual conformational states of the receptor‒G protein interface.

This comparative analysis reveals the strengths and limitations of current AI/ML tools in structural predictions of GPCRs. While the overall structural predictions were highly accurate, the finer details of the ligand conformation and receptor‒G protein interactions were not completely captured by the AI models. These differences are crucial for understanding the functional dynamics of receptors and have significant implications for drug design.

## Discussion

In this study, we have advanced our understanding of the structural mechanisms underlying the activation of MCHR1‒G_i_ and MCHR2‒G_q_ by the endogenous ligand MCH. Our approach, integrating high-resolution structural determination, mutagenesis, and functional assays, has provided a detailed view of the ligand-binding sites in MCHR1 and MCHR2. We highlight the crucial role of the ring structure formed by Cys6 and Cys17 in ligand‒receptor interactions.

Our comparative analysis of the ligand-binding pockets in MCHR1 and MCHR2 unveils unique molecular interactions. Notably, the absence of a hydrogen bond between S^7.42^ and R11 in MCHR1, due to the presence of G^7.42^, contrasts with the hydrogen bond formation in MCHR2. Additionally, the differences in steric hindrance between W^6.48^ in MCHR1 and A^6.48^ in MCHR2 lead to distinct displacement and interaction patterns with the ligand. These differences are further illustrated by the unique contacts MCH makes with MCHR2, facilitated by the spatial arrangement of W^2.64^ and Y^7.32^.

The activation process in both MCHR1 and MCHR2 involves significant conformational changes in the canonical motifs DRY, NPxxY, and PIF, indicative of receptor activation. However, we observe a divergence from the typical toggle switch mechanism seen in class A GPCRs. In MCHR1, W^6.48^ shows minimal movement after activation, whereas MCHR2 has an alanine at this key position. Importantly, the disruption of the polar interaction between Q^3.36^ and H/Y^6.52^ in MCHR1, as evidenced by our mutagenesis studies, plays a pivotal role in receptor activation. These findings are complemented by the differential effects of mutations at these positions in MCHR1 and MCHR2, underlining the unique activation mechanisms in these receptors.

Furthermore, our structural insights extend to the interactions between MCHRs and G proteins. The T^34.54^R mutation in the ICL2 of MCHR1, identified through our mutagenesis studies, significantly enhances Gα_q_ coupling efficiency. This observation emphasizes the role of specific residues in determining G protein coupling specificity. However, a similar mutation in MCHR2 does not yield an increase in Gα_q_ coupling efficiency, highlighting the complexity of GPCR‒G protein interactions and the necessity to consider receptor-specific structural and functional nuances.

Additionally, our comparative analysis with AlphaFold2 predictions has demonstrated both the capabilities and limitations of AI/ML tools in structural biology. While AlphaFold2 accurately predicted the overall receptor architectures, it fell short in capturing the precise conformation of ligands and the subtle yet functionally significant movements in receptor domains and associated proteins. This comparison underscores the importance of combining computational predictions with experimental methods to achieve a comprehensive understanding of complex biological systems like GPCRs.

Overall, our study provides a rich and comprehensive understanding of the molecular dynamics in ligand binding, receptor activation, and G protein coupling in MCHR1 and MCHR2. These findings pave the way for targeted therapeutic interventions, leveraging the distinct characteristics of these receptors.

## Materials and methods

### Constructs cloning

The gene sequences encoding human MCHR1 (residues 70‒405) and MCHR2 (residues 1‒331, with L256^6.40^Y mutation) were subcloned into pFastBac vector (Invitrogen) with an N-terminal haemagglutinin signal peptide (HA) and a thermostabilized BRIL fusion, followed by a C-terminal 15-amino acid linker, a LgBiT subunit and two MBPs to facilitate receptor expression and stability. The Gα_i_ construct was designed with dominant-negative mutations S47N, G203A, A326S, and E245A to decrease the affinity of nucleotide-binding and stable Gαβγ complex. The mini-Gαq construct was designed with the C-terminal α-helix (αH5) of Gαq, which is critical for G protein coupling selectivity. The core of the construct, known as the Ras domain, is derived from Gαs. This design choice was made to enable the binding of the nanobody Nb35, which stabilizes the complex for cryo-EM analysis. Additionally, the N-terminal helix from Gαi was incorporated into the construct to facilitate the binding of the single-chain variable fragment (scFv16), aiding in cryo-EM studies. Rat Gβ1 was constructed with a N-terminal 16× His-tag for purification and a C-terminal HiBiT to form a NanoBiT with LgBiT. Gα_i_, mini-Gα_q_, Gβ1, Gγ2, scFv16, and Nb35 were all cloned into the pFastBac vector separately. The wild-type and mutants of MCHR1 and MCHR2 were constructed into the pcDNA6.0 vector (Promega) for functional assays.

### Complex expression and purification

MCHRs and G proteins were co-expressed in High Five insect cells (Thermo Fisher Scientific) using Bac-to-Bac baculovirus system (Thermo Fisher Scientific). The cells were cultured in ESF 921 serum-free medium (Expression Systems) to a density of 3 × 10^6^ cells/mL. The cells were infected with MCHR1, Gα_i_, Gβ_1_, Gγ_2_, scFv16, and Ric8B at the ratio of 1:2:2:2:2:2 or infected with MCHR2, Gα_q_, Gβ_1_, Gγ_2_, and Ric8A at the ratio of 1:2:2:2:2. After 48 h of infection at 27 °C; the cells were harvested by centrifugation at 2000 rpm and then stored at ‒80 °C for future use.

The frozen cells were thawed at room temperature (RT) and resuspended in a buffer containing 20 mM HEPES, pH 7.4, 100 mM NaCl, 10% (v/v) glycerol, 10 mM MgCl_2_, 10 mM CaCl_2_, 100 μM TCEP, 25 mU/mL apyrase, 1× protease inhibitor cocktail (TargetMol, 1 mL/100 mL suspension), 10 μM MCH and 10 μg/mL Nb35 (for MCHR2 only). The suspension was incubated at RT for 1 h and then solubilized by 0.5% (w/v) lauryl maltose neopentyl glycol (LMNG, Anatrace) and 0.1% (w/v) cholesteryl hemisuccinate TRIS salt (CHS, Anatrace) at 4 °C for 3 h. The supernatant was separated by centrifugation at 30,000 rpm for 35 min and then incubated with re-equilibrated dextrin beads (Smart-Lifesciences) at 4 °C for 3 h. The resin was collected by centrifugation at 500× *g* for 5 min, loaded onto a gravity-flow column and washed with 20 column volumes of wash buffer containing 20 mM HEPES, pH 7.4, 100 mM NaCl, 10% (v/v) glycerol, 5 mM MgCl_2_, 5 mM CaCl_2_, 25 μM TCEP, 10 μM MCH, 0.01% (w/v) LMNG, 0.01% (w/v) glyco-diosgenin (GDN, Anatrace) and 0.004% (w/v) CHS. The protein was eluted with wash buffer adding 10 mM maltose, concentrated using a 100-kDa Amicon Ultra Centrifugal Filter (Millipore), and loaded onto Superose 6 Increase 10/300 GL column (GE Healthcare) pre-equilibrated with buffer including 20 mM HEPES, pH 7.4, 100 mM NaCl, 2 mM MgCl_2_, 2 mM CaCl_2_, 100 μM TCEP, 0.0005% (w/v) digitonin (Biosynth), 0.00075% (w/v) LMNG, 0.00025% (w/v) GDN, and 0.0002% (w/v) CHS. The receptor and G protein complexes were collected and concentrated for electron microscopy experiments.

### Expression and purification of Nb35

Nanobody-35 (Nb35) was expressed in *E. coli* BL21 cells which were cultured in TB medium with 100 μg/mL ampicillin at 37 °C for about 3 h to OD_600_ of 1.0 and then induced with 1 mM IPTG at 25 °C for 16 h. Each liter of harvested cells was resuspended with 15 mL TES (0.2 M Tris, pH 8.0, 0.5 mM EDTA, 0.5 M sucrose), stirred at 4 °C for 1 h and another 45 min with 30 mL TES/4. The supernatant was collected by centrifugation at 30,000 rpm for 30 min and incubated with pre-equilibrated Nickel resin at 4 °C for 1 h. After washing with 20 column volumes of wash buffer containing 20 mM HEPES, pH 7.4, 100 mM NaCl, and 10% (v/v) glycerol, Nb35 was eluted with wash buffer, adding 300 mM imidazole. The protein was further purified by HiLoad 16/600 Superdex 75 column with 20 mM HEPES pH 7.4, and 100 mM NaCl. The targeted fractions were collected and concentrated to 5 mg/mL and then flash-frozen in liquid nitrogen before storing at −80 °C.

### Cryo-EM grid preparation and data collection

For cryo-EM grid preparation of the MCH‒MCHR1‒G_i_ complex, 3 μL of the purified complex at a concentration of 5.72 mg/mL was applied to glow-discharged holey carbon grids (Quantifoil R1.2/1.3, Au/C 300 mesh) that were subsequently vitrified by plunging into liquid ethane using a Vitrobot Mark IV (Thermo Fisher Scientific) at 4 °C. A Titan Krios G4 equipped with a Gatan K3 direct electron detector with super-resolution mode and EPU was used to acquire cryo-EM movies at the Advanced Center for Electron Microscopy at Shanghai Institute of Materia Medica, Chinese Academy of Sciences. A total of 14,952 movies were recorded with a pixel size of 0.824 Å at a dose of 50 electrons per Å^2^ for 32 frames. The defocus range of this dataset was ‒0.8 to ‒1.8 μm.

For the MCH‒MCHR2‒G_q_ complex, 20.47 mg/mL of purified protein was used for cryo-EM grid preparation. Cryo-EM movies were collected by a Titan Krios G4 CEFG at 300 kV accelerating voltage equipped with Falcon4 direct electron detector and Selectris X (Thermo Fisher Scientific). A total of 8789 EER movies were recorded with a pixel size of 0.73 Å at a dose of 50 electrons per Å^2^. The defocus range of this dataset was ‒0.8 to ‒1.8 μm.

### Cryo-EM data processing and three-dimensional reconstruction

For the MCH‒MCHR1‒G_i_ complex, all dose-fractionated image stacks were subjected to beam-induced motion correction by Relion4.0^41^. The defocus parameters were estimated by CTFFIND4.1^42^. Blob picking and Template picking yielded 32,120,445 particles, which were processed by reference-free 2D classification using Cryosparc^43^. With the initial model from Cryosparc, after several rounds of 3D classification using Relion, 311,033 particles were used to further CTF Refinement and polishing, yielding a reconstruction with a global resolution of 3.01 Å at a Fourier shell correlation (FSC) of 0.143, and subsequently post-processed by DeepEMhancer^44^.

For the MCH‒MCHR2‒G_q_ complex, EER movies were aligned with Relion4.0^41^. Initial contrast transfer function (CTF) fitting was performed with CTFFIND4.1^42^ from Cryosparc^43^. Blob picking and Template picking yielded 14,206,080 particles, which were processed by reference-free 2D classification using Cryosparc, 2D Classification was processed using Cryosparc, producing 1,498,863 particles for further processing. With the initial model, two rounds of 3D classifications were carried out with Relion4.0, in which 879,702 particles were subjected to 3D auto-refinement, CTF refinement, and polishing. A map with an indicated global resolution of 2.4 Å at FSC of 0.143 was generated from the final 3D refinement and subsequently post-processed by DeepEMhancer^44^.

### Model building and refinement

All PDB coordinates using alphafold2^45^ were served as a starting model for building the atomic model. All model were fitted into cryo-EM density map using chimera^46^ followed by a manual adjustment in Coot^47^. The model was refined by Phenix^48^.

### GloSensor cAMP assay

GloSensor cAMP assay was used to detect the downstream Gα_i_ signal of MCHR1 using GloSensor cAMP Reagent (Promega). HEK293 cells were cultured in DMEM/high glucose supplemented with 10% (v/v) fetal bovine serum and 1% (v/v) penicillin–streptomycin at 37 °C in 5% CO_2_. Cells were grown at 12-well plates for 18 h and transfected with MCHR1 constructs and the cAMP biosensor GloSensor-22F (Promega) at a ratio of 3:2. After transfection for 24 h, cells were harvested, resuspended with CO_2_-independent media containing 2% GloSensor cAMP Reagent (Promega), and then distributed to 384-well plates at a density of 3 × 10^5^ cells/mL (20 μL/well). The cells were incubated at 37 °C for 1.5 h. Ligand of different concentrations was mixed with forskolin at the final concentration of 1 μM (Sigma), and the mixture was added to each well (10 μL/well). The sample mixtures were immediately measured with an EnVision multi-plate reader (PerkinElmer).

### Inositol phosphate accumulation assay

IP1 accumulation assay was applied for the detection of the downstream Gα_q_ signal of MCHR2 using the IP-One HTRF kit (Cisbio). AD293 cells were cultured in DMEM/high glucose supplemented with 10% (v/v) fetal bovine serum and 1% (v/v) penicillin–streptomycin at 37 °C in 5% CO_2_. After transfection for 24 h, cells were harvested, resuspended with 1× stimulation buffer to a density of 8 × 10^5^ cells/mL, and then seeded to 384-well plates for 7 μL/well. After dispensing 7 μL different concentrations of ligand diluted with 1× stimulation buffer, the mixture was incubated at 37 °C for 1 h. IP1-d2 and anti-IP1 cryptate were dissolved in 1× lysis buffer and sequentially added to the plates for 3 μL/well. Before measurement, the samples were incubated at RT for 30 min and measured with EnVision multi-plate reader (PerkinElmer).

### Cell surface expression assay

MCHR1/2 wild type and their mutants were cloned into pcDNA6.0 (Invitrogen) with an N-terminal 3× Flag tag, and the cell surface expression was determined by flow cytometry. The expression of MCHR1 was tested by HEK293, and the expression of MCHR2 was measured by AD293. After transfection for 24 h, cells were collected and then blocked with 50 μL 5% (w/v) bovine serum albumin (BSA) in PBS at RT for 15 min, followed by incubation with primary mouse anti-Flag antibody (ABclonal) at RT for 1 h. The cells were then washed three times with 1 mL 1% BSA (w/v) and incubated with anti-mouse Alexa-488-conjugated secondary antibody (Invitrogen) at 4 °C in the dark for 1 h. After another three washes with 1 mL 1% BSA (w/v), cells were resuspended with 200 μL 1% BSA (w/v) for detection in Guava EasyCyte™ System at excitation 488 nm and emission 525 nm.

### Alphafold2 multimer prediction

The 2.3.2 version of AlphaFold 2 multimer version^49,50^ was applied for the prediction of MCH‒MCHR1‒G_i_ and MCH‒MCHR2‒G_q_ structures. The input sequences and chain IDs are the same as the cryo-EM structures. The template date was 2021-11-01, and the full database present was applied. The prediction was run on a Tesla V100 GPU.

### Quantification and statistical analysis

All functional study data were analyzed using Prism 8 (GraphPad) and displayed as means ± SEM from at least three independent experiments. Concentration–response curves were evaluated with a three-parameter logistic equation. The significance was determined with one-way ANOVA with Tukey’s test, and *P* < 0.05 vs wild type was considered statistically significant.

### Supplementary information

Supplementary information

## Data Availability

Cryo-EM maps have been deposited in the Electron Microscopy Data Bank under accession codes: EMD-37823 (MCH‒MCHR1‒G_i_ complex) and EMD-37824 (MCH‒MCHR2‒G_q_ complex). The atomic coordinates have been deposited in the Protein Data Bank under accession codes: 8WSS (MCH‒MCHR1‒G_i_ complex) and 8WST (MCH‒MCHR2‒G_q_ complex).
