# Supplementary material for: Mechanisms of ligand recognition and activation of melanin-concentrating hormone receptors
Source: Cell Discov. 2024 May 7;10:48. doi: 10.1038/s41421-024-00679-8 (PMC11074101; doi:10.1038/s41421-024-00679-8)
Supplement: Supplementary file 1 — Supplementary information [file 41421_2024_679_MOESM1_ESM.pdf]

Supplementary Information

Mechanisms of ligand recognition and activation of melanin-concentrating hormone receptors

Qian He, Qingning Yuan, Hong Shan, Canrong Wu, Yimin Gu, Kai Wu, Wen Hu, Yumu Zhang, Xinheng He, H. Eric Xu, and Li-Hua Zhao

Table of contents

Supplementary Fig. S1 | MCH-MCHR1-G_i complexes purification and cryo-EM data processing. Related to Figure 1.

Supplementary Fig. S2 | MCH-MCHR2-G_q complexes purification and cryo-EM data processing. Related to Figure 1.

Supplementary Fig. S3 | Density maps of MCH-MCHR1-G_i and MCH-MCHR2-G_q complexes. Related to Figure 1.

Supplementary Fig. S4 | Mutagenesis data of key residues in the ligand binding pockets of MCHR1 and MCHR2. Related to Figure 2 and 3.

Supplementary Fig. S5 | Sequence alignment of the key MCH-binding residues between MCHR1 and MCHR2. Related to Figure 2, 3 and Supplementary Fig.4.

Supplementary Fig. S6 | Sequence alignment of MCHR1, MCHR2, SSTR2 and V2R. Related to Figure 7.

Supplementary Fig. S7 | Conformational comparison of AlphaFold2 models with cryo-EM structures.

Supplementary Table S1. Cryo-EM data collection, model refinement and validation statistics.

Supplementary Table S2. Interaction of MCH with key residues in the ligand-binding pockets of MCHR1.

Supplementary Table S3. Interaction of MCH with key residues in the ligand-binding pockets of MCHR2.

Supplementary Table S4. Comparison of crucial residues within the ligand-binding pockets between MCHR1 and MCHR2.

Supplementary Table S5. *p*EC50 values, Emax and the expression level of mutations of MCH-binding pocket in MCHR1.

Supplementary Table S6. *p*EC50 values, Emax and the expression level of mutations of MCH-binding pocket in MCHR1.

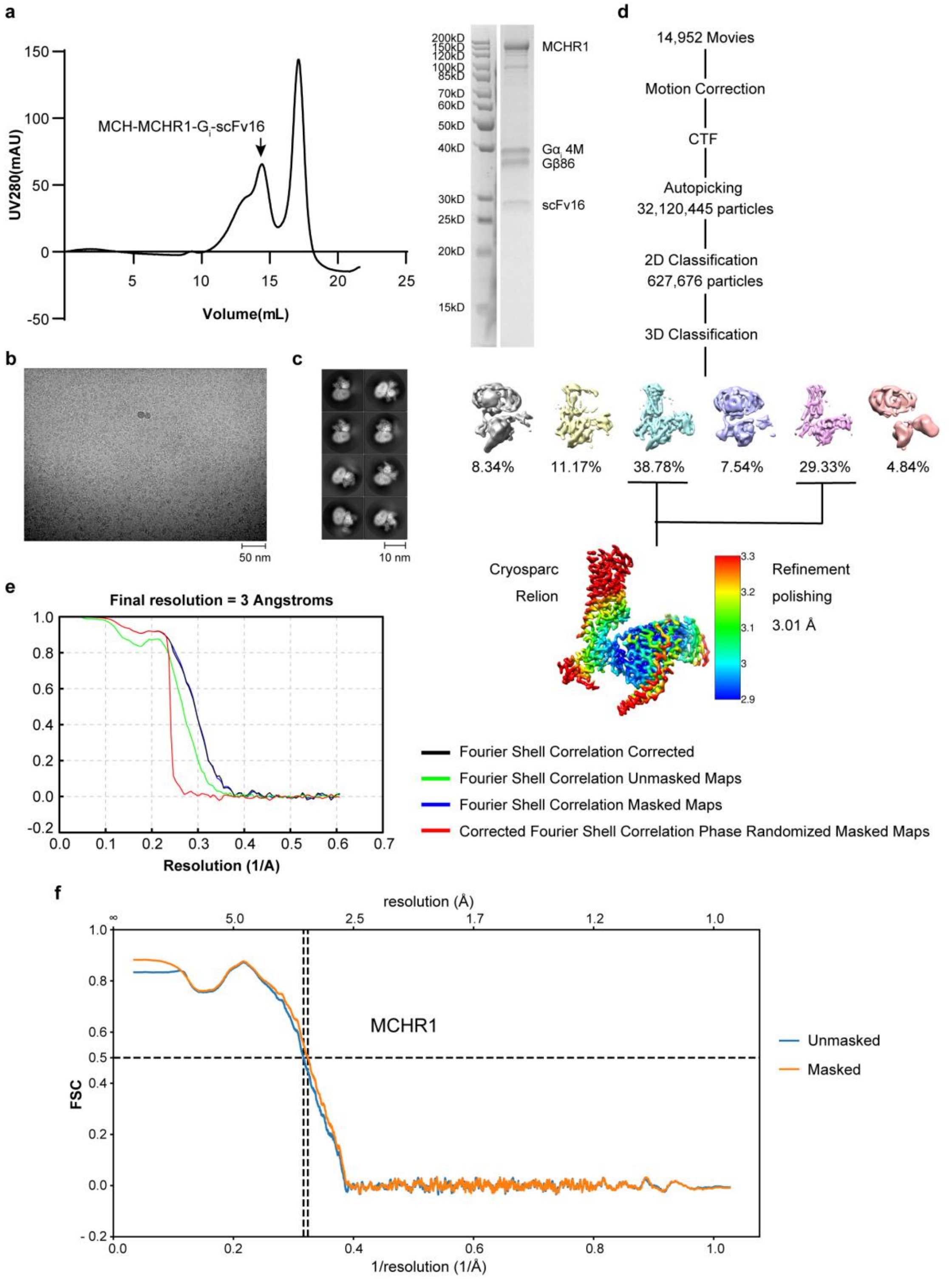

Supplementary Fig. S1 | MCH-MCHR1-G_i complexes purification and cryo-EM data processing. Related to Figure 1. a, Profiles of size-exclusion chromatography elution (left) and SDS-PAGE analysis (right) of MCH-MCHR1-G_i complexes. Black arrow refers to complex monomer. **b,c,** Representative cryo-EM images (**b**) and 2D classification (**c**) of the MCH-MCHR1-G_i complexes. **d,** Workflow of cryo-EM single particle analysis of the MCH-MCHR1-G_i complexes.

e, Fourier shell correlation curves of the MCH-MCHR1-G_i complexes. **f,** Fourier shell correlation curves between model and map of MCH-MCHR1-G_i complexes using Phenix.

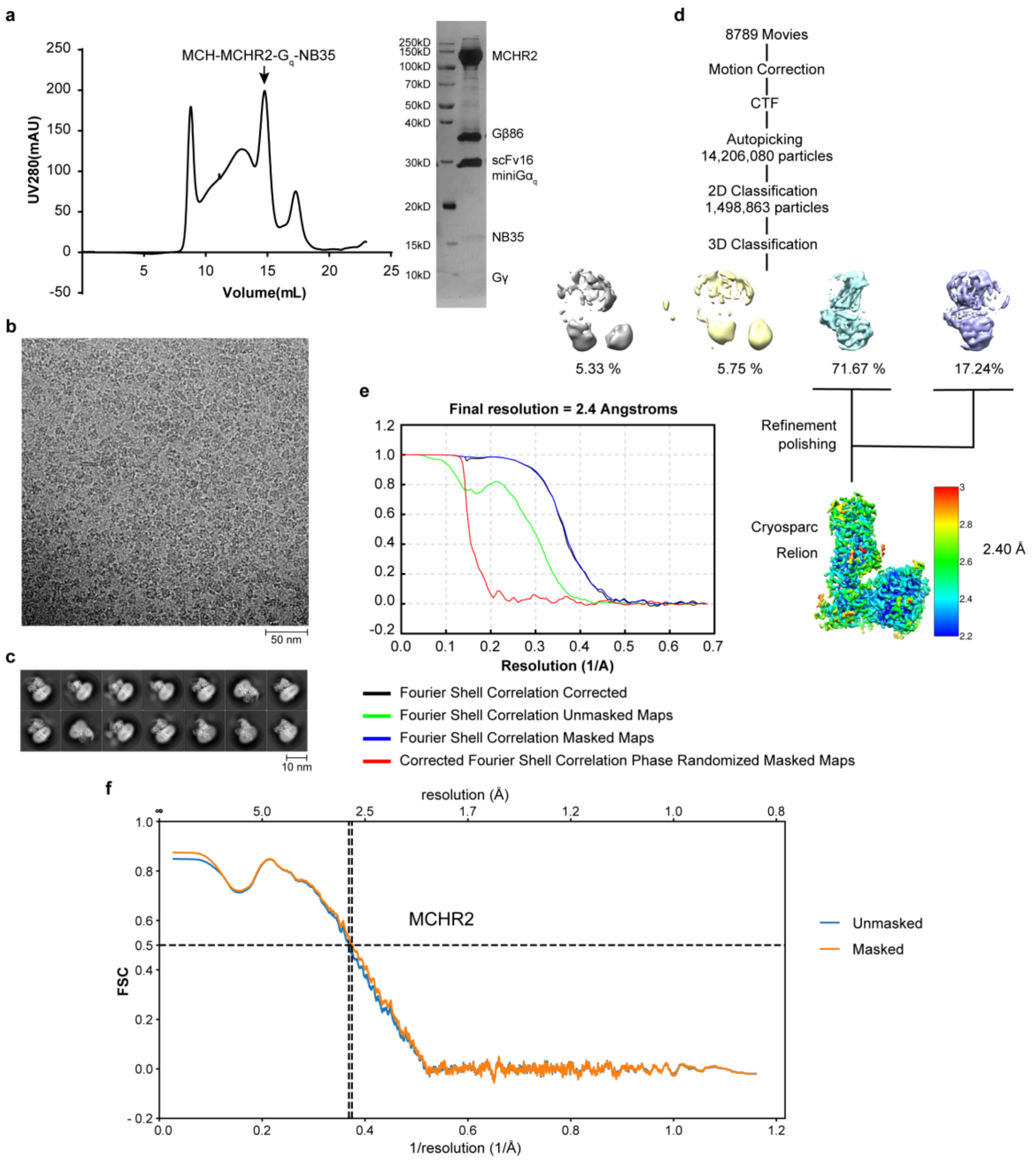

Supplementary Fig. S2 | MCH-MCHR2-G$_q$ complexes purification and cryo-EM data processing. Related to Figure 1. a, Profiles of size-exclusion chromatography elution (left) and SDS-PAGE analysis (right) of MCH-MCHR2-G$_q$ complexes. Black arrow refers to complex monomer. **b,c,** Representative cryo-EM image (**b**) and 2D classification (**c**) of the MCH-MCHR2-G$_q$ complexes. **d,** Workflow of cryo-EM single particle analysis of the MCH-MCHR2-G$_q$ complexes. **e,** Fourier shell correlation curves of the MCH-MCHR2-G$_q$ complexes. **f,** Fourier shell correlation curves between model and map of MCH-MCHR2-G$_q$ complexes using Phenix.

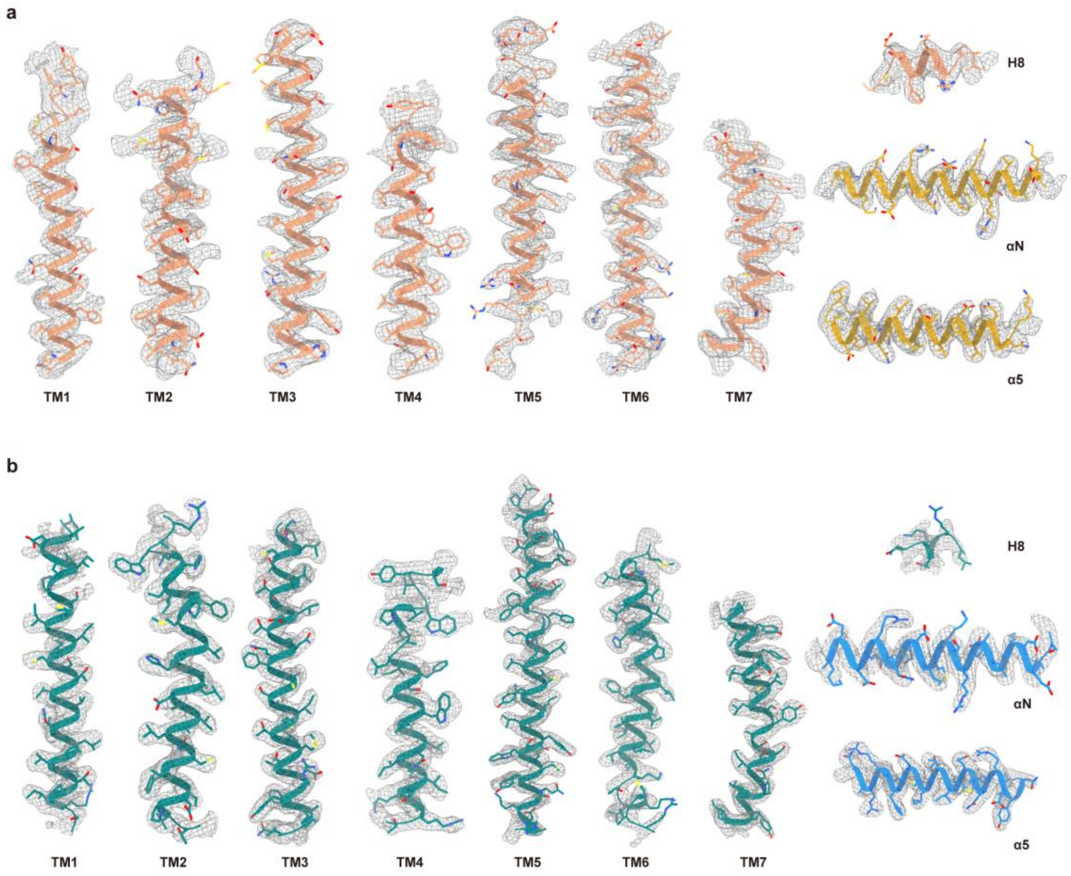

Supplementary Fig. S3 | Density maps of MCH-MCHR1-G_i and MCH-MCHR2-G_q complexes. Related to Figure 1. a, Depicts the representative resolution map of TM1–TM7 and helix 8 of MCHR1, as well as the αN helix and α5 helix of $G\alpha_i$ within the MCH-MCHR1-G_i complexes. **b,** Depicts the representative resolution map of TM1–TM7 and helix 8 of MCHR2, along with the αN helix and α5 helix of $G\alpha_q$ within the MCH-MCHR2-G_q complexes.

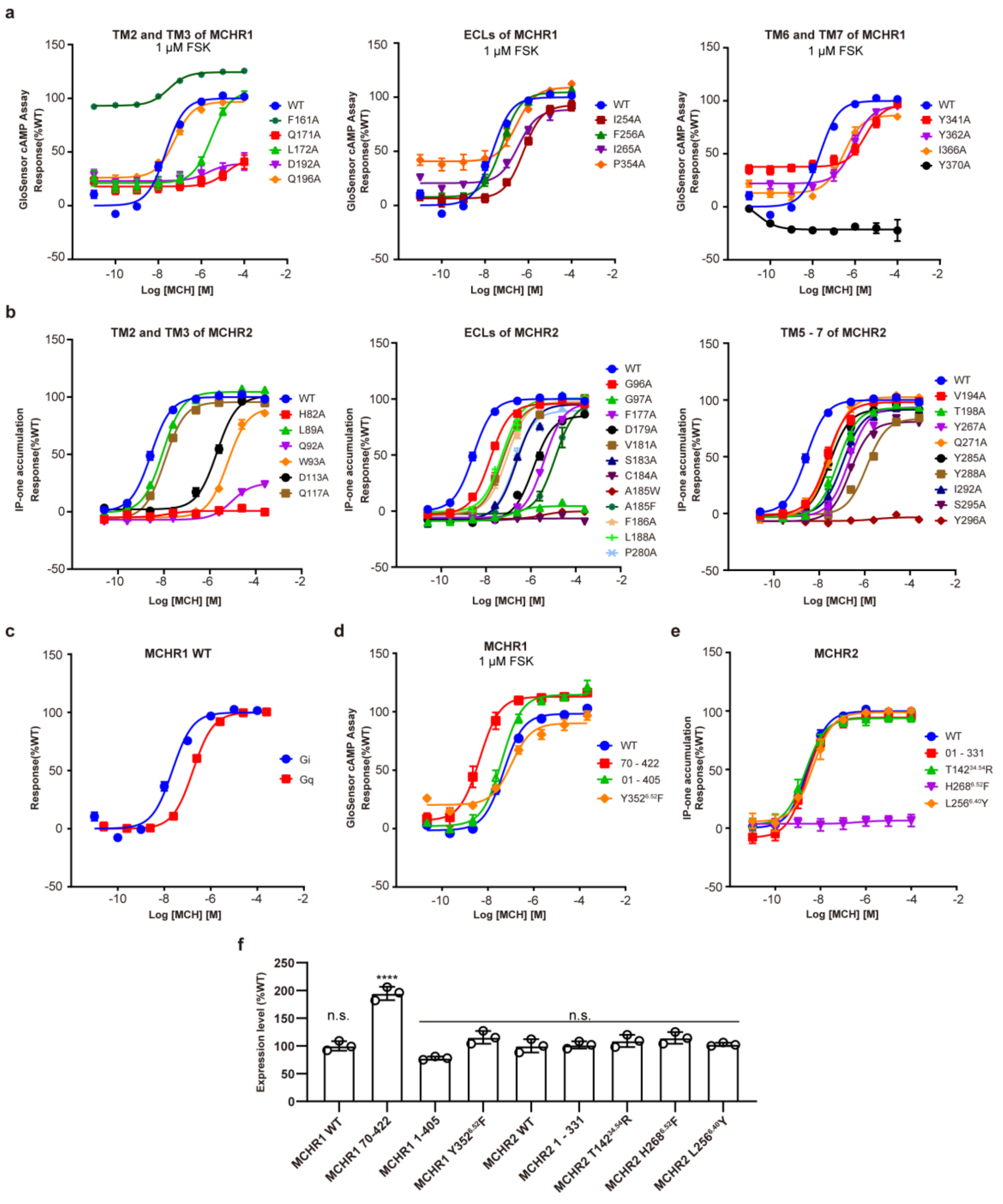

Supplementary Fig. S4 | Mutagenesis data of key residues in the ligand binding pockets of MCHR1 and MCHR2. Related to Figure 2 and 3. a, Effects of mutations in the binding pocket of MCHR1 by Glo-sensor cAMP assay. **b,** Effects of mutations in the binding pocket of MCHR2 by IP-one accumulation assay. **c,** Comparison of the efficacy of MCH-activated MCHR1 in coupling with G_i and G_q. **d,e,** Effects of mutations and truncations of MCHR1 and MCHR2. **f,** The expression level of mutations and truncations of MCHR1 and MCHR2.The response data was normalized by WT receptor within each individual experiment. Data from three independent experiments, each of which was performed in triplicate, are presented as mean ± S.E.M. Statistical differences were

determined by two-sided one-way ANOVA with Tukey's test compared with WT. n.s. no significant difference; ****$P < 0.0001$.

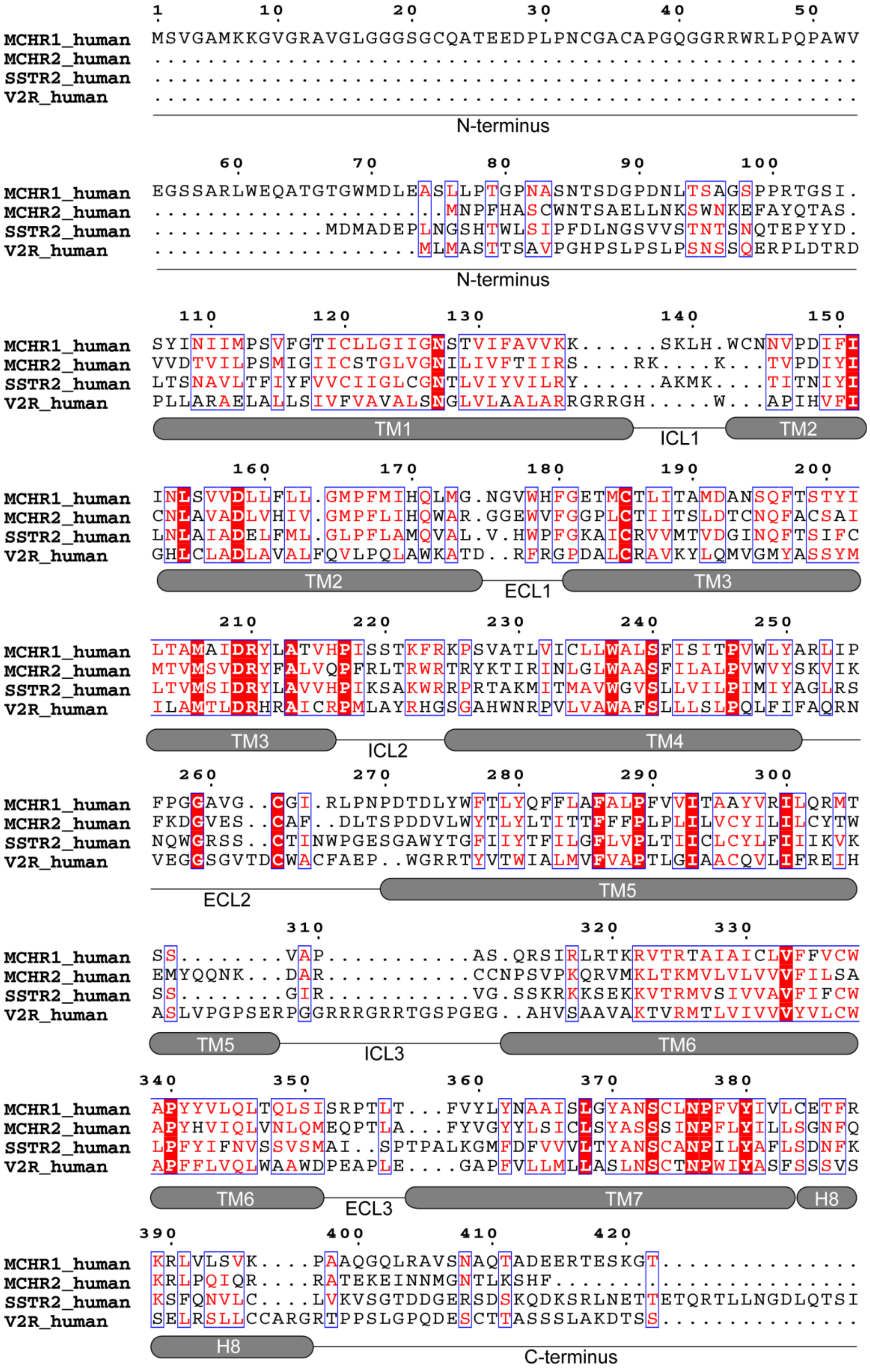

Supplementary Fig. S5 | Sequence alignment of the key MCH-binding residues between

MCHR1 and MCHR2. Related to Figure 2, 3 and Supplementary Fig.4.

The sequence alignment of the key MCH-binding residues between MCHR1 and MCHR2 was created by ClustalW website and the graphic was drawn by the ESPript 3.0 website. The key MCH-binding residues of MCHR1 was indicated by the light-salmon circles and the key MCH-binding residues of MCHR2 was pointed by the teal triangles. TM1-7 and helix8 are shown by columns under the sequences. Colors represent the similarity of residue: red background, identical; red text, strongly similar.

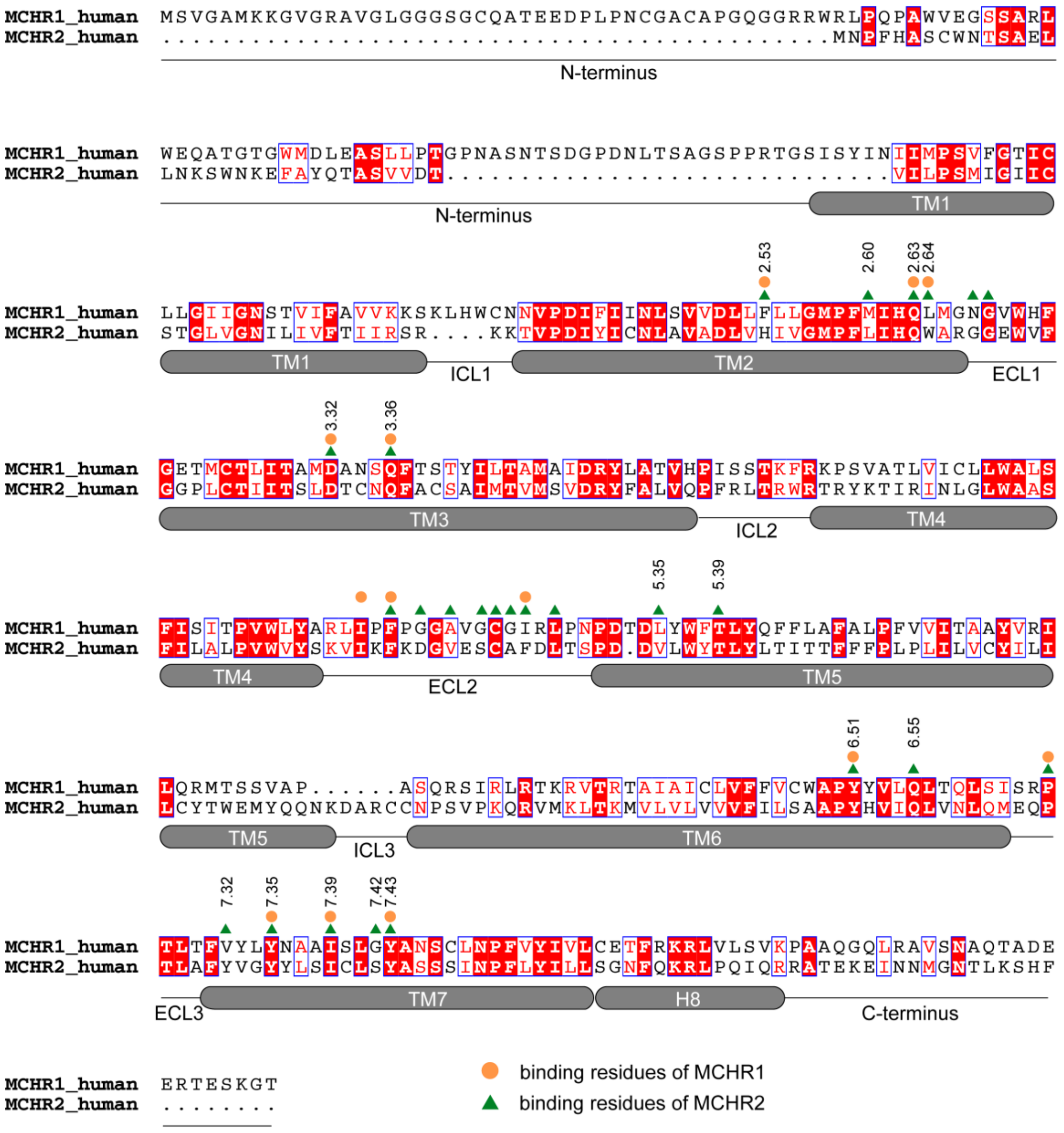

Supplementary Fig. S6 | Sequence alignment of MCHR1, MCHR2, SSTR2 and V2R. Related to Figure 7.

The sequence alignment of MCHR1, MCHR2, SSTR2 and V2R was created by GPCR databank and the graphic was drawn on the ESPript 3.0 website. TM1-7 and helix8 are shown by columns under the sequences. Colors represent the similarity of residue: red background, identical; red text, strongly similar.

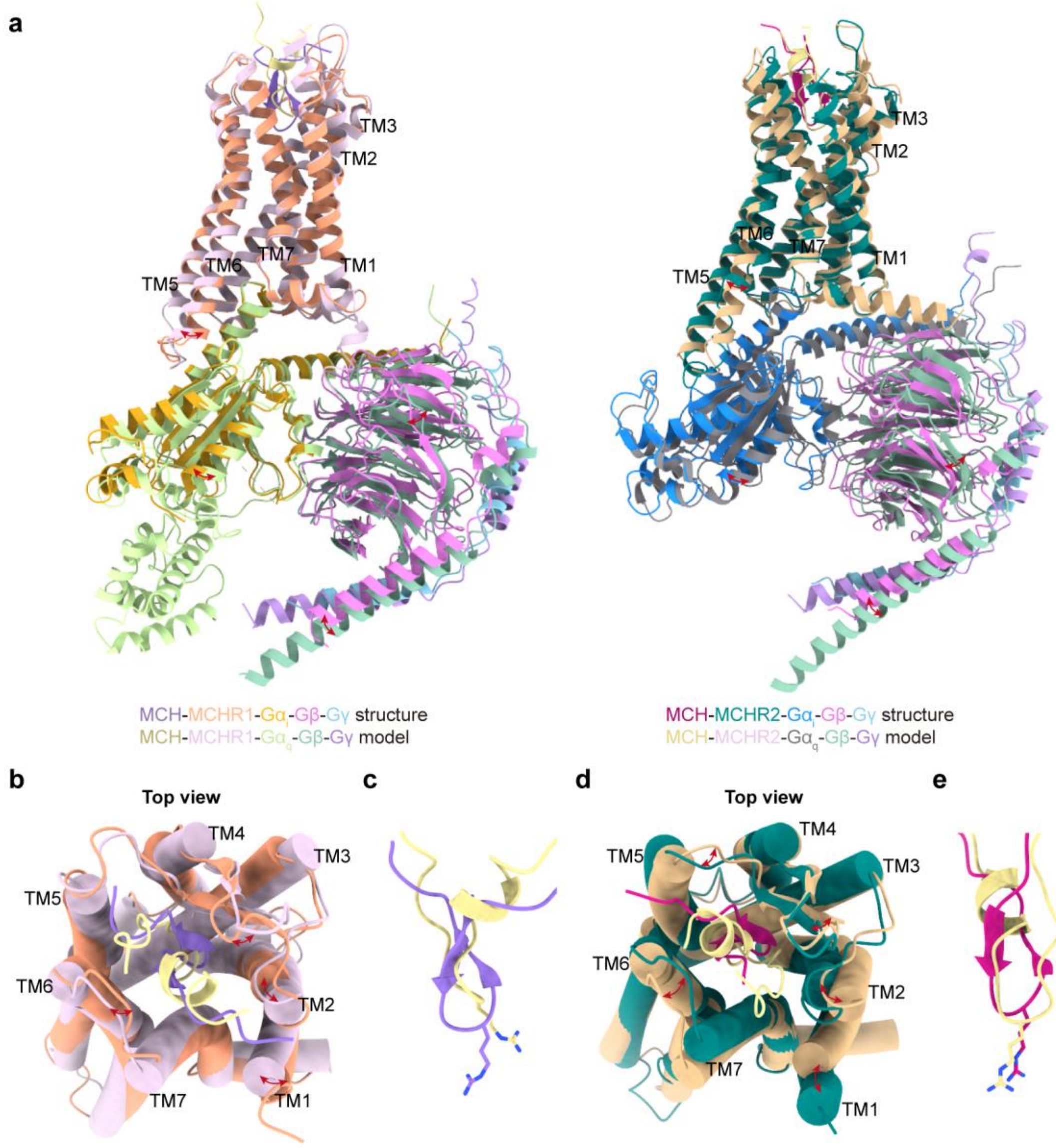

Supplementary Fig. S7 | Conformational comparison of AlphaFold2 models with cryo-EM structures. a, Structural superposition of the AlphaFold2-predicted models and the MCH-activated MCHR1 and MCHR2 cryo-EM structures. **b, d,** Top view of the structural alignment between AlphaFold2-predicted models and cyro-EM structures of MCHR1(**b**) and MCHR2(**d**).**c, e,** Comparison of two ligands from AlphaFold2 models with cryo-EM structures of MCHR1(**c**) and MCHR2(**e**).

Supplementary Table S1. Cryo-EM data collection, model refinement and validation statistics.

	MCHR1	MCHR2
**Data collection and processing**		
Magnification	105K	165K
Voltage (kV)	300	300
Electron exposure ($e^-/Å^2$)	50	50
Defocus range (μm)	-0.8 to -1.8	-0.8 to -1.8
Pixel size (Å)	0.824	0.73
Symmetry imposed	C1	C1
Initial particle images (no.)	32,120,445	14,206,080
Final particle images (no.)	393,333	879,702
Map resolution (Å)		
FSC threshold	0.143	0.143
Map resolution (Å)	3.01	2.40
**Refinement**		
Model resolution (Å)	3.1	2.7
FSC threshold	0.5	0.5
Model-Map CC (mask)	0.84	0.75
**Model composition**		
Non-hydrogen atoms	8814	8287
Protein residues	1147	1055
B factors ($Å^2$)		
Protein	53.96	33.83
R.m.s. deviations		
Bond lengths (Å)	0.002	0.002
Bond angles (Å)	0.4463	0.449
**Validation**		
MolProbity score	1.43	1.44
Clash score	5.16	4.91
Rotamer outliers (%)	0.32	0.22
Ramachandran plot		
Favored (%)	97.16	96.93
Allowed (%)	2.84	3.07
Disallowed (%)	0	0

Supplementary Table S2. Interaction of MCH with key residues in the ligand-binding pockets of MCHR1.

MCH	MCHR1	Interactions
Leu5	$P354^{ECL3}$	Hydrophobic interaction
Arg6	$F256^{ECL2}$	Hydrophobic interaction
	$P354^{ECL3}$	
Met8	$L172^{2.64}$	Hydrophobic interaction
	$Y362^{7.35}$	
	$I366^{7.39}$	
Leu9	$Y362^{7.35}$	Hydrophobic interaction
Gly10	$Y341^{6.51}$	Hydrophobic interaction
	$Y362^{7.35}$	
Arg11	$F161^{2.53}$	Hydrophobic interaction
	$D192^{3.32}$	Electrostatic interaction
	$Q196^{3.36}$	Side chain-side chain hydrogen bond
	$Y370^{7.43}$	Cation−π interaction
Val12	$I265^{ECL2}$	Hydrophobic interaction
Tyr13	$Q171^{2.63}$	Backbone-side chain hydrogen bond
	$L172^{2.64}$	Hydrophobic interaction
Pro15	$I254^{ECL2}$	Hydrophobic interaction

Supplementary Table S3. Interaction of MCH with key residues in the ligand-binding pockets of MCHR2.

MCH	MCHR2	Interactions
Leu5	$F177^{ECL2}$	Hydrophobic interaction
Arg6	$D179^{ECL2}$	Electrostatic interaction
	$V181^{ECL2}$	Hydrophobic interaction
	$G96^{ECL1}$	Side chain-backbone hydrogen bond
Met8	$W93^{2.64}$	Hydrophobic interaction
	$P280^{ECL3}$	
	$Y285^{7.32}$	
	$Y288^{7.35}$	
Leu9	$F186^{ECL2}$	Hydrophobic interaction
	$L188^{ECL2}$	
	$V194^{5.35}$	
	$T198^{5.39}$	
	$Y288^{7.35}$	
Gly10	$Q271^{6.55}$	Backbone-side chain hydrogen bond
	$Y267^{6.51}$	Hydrophobic interaction
	$Y288^{7.35}$	
Arg11	$H82^{2.53}$	Hydrophobic interaction
	$W93^{2.64}$	Backbone-side chain hydrogen bond
	$D113^{3.32}$	Electrostatic interaction
	$Q117^{3.36}$	
	$Y288^{7.35}$	Hydrophobic interaction
	$I292^{7.39}$	
	$S295^{7.42}$	Side chain-side chain hydrogen bond
	$Y296^{7.43}$	Cation−π interaction
Vla12	$L89^{2.60}$	Hydrophobic interaction
	$C184^{ECL2}$	
	$F186^{ECL2}$	
Tyr13	$Q92^{2.63}$	Hydrophobic interaction
	$W93^{2.64}$	
	$G97^{ECL1}$	
	$S183^{ECL2}$	
	$C184^{ECL2}$	
	$A185^{ECL2}$	
	$F186^{ECL2}$	Backbone-backbone hydrogen bond
	$Y285^{7.32}$	Side chain-side chain hydrogen bond
Pro15	$F186^{ECL2}$	Hydrophobic interaction
	$L188^{ECL2}$	

Supplementary Table S4. Comparison of crucial residues within the ligand-binding pockets between MCHR1 and MCHR2.

MCH	MCHR1	MCHR2
Leu5	$P354^{ECL3}$	$F177^{ECL2}$
Arg6	$F256^{ECL2}$	$D179^{ECL2}$
	$P354^{ECL3}$	$V181^{ECL2}$
		$G96^{ECL1}$
Met8	$L172^{2.64}$	$W93^{2.64}$
		$P280^{ECL3}$
	$Y362^{7.35}$	$Y288^{7.35}$
	$I366^{7.39}$	$Y285^{7.32}$
Leu9		$F186^{ECL2}$
		$L188^{ECL2}$
		$V194^{5.35}$
		$T198^{5.39}$
	$Y362^{7.35}$	$Y288^{7.35}$
Gly10	$Y341^{6.51}$	$Y267^{6.51}$
		$Q271^{6.55}$
	$Y362^{7.35}$	$Y288^{7.35}$
Arg11	$F161^{2.53}$	$H82^{2.53}$
		$W93^{2.64}$
	$D192^{3.32}$	$D113^{3.32}$
	$Q196^{3.36}$	$Q117^{3.36}$
		$Y288^{7.35}$
		$I292^{7.39}$
		$S295^{7.42}$
	$Y370^{7.43}$	$Y296^{7.43}$
Val12		$L89^{2.60}$
		$C184^{ECL2}$
	$I265^{ECL2}$	$F186^{ECL2}$
Tyr13	$Q171^{2.63}$	$Q92^{2.63}$
	$L172^{2.64}$	$W93^{2.64}$
		$G97^{ECL1}$
		$S183^{ECL2}$
		$C184^{ECL2}$
		$A185^{ECL2}$
		$F186^{ECL2}$
		$Y285^{7.32}$
Pro15	$I254^{ECL2}$	$F186^{ECL2}$
		$L188^{ECL2}$

Supplementary Table S5. ***p*****EC50 values, Emax and the expression level of mutations of MCH-binding pocket in MCHR1.**

MCHR1 + MCH				
**Mutant**	***p*EC$_{50}$ ± S.E.M.**	**Emax (% WT) ± S.E.M**	***P* Value**	**Expression (%WT) ± S.E.M**
WT	7.62 ± 0.07	100	> 0.9999	100
F161$^{2.53}$A	7.52 ± 0.01	124 ± 1	> 0.9999	120 ± 2
Q171$^{2.63}$A	4.93 ± 0.13	57 ± 6	< 0.0001	103 ± 3
L172$^{2.64}$A	5.51 ± 0.11	107 ± 3	< 0.0001	93 ± 3
D192$^{3.32}$A	5.51 ± 0.34	40 ± 7	< 0.0001	120 ± 2
Q196$^{3.36}$A	7.24 ± 0.16	97 ± 1	0.5299	120 ± 6
I254^{ECL2}A	6.22 ± 0.11	98 ± 3	<0.0001	100 ± 3
F256^{ECL2}A	7.22 ± 0.06	106 ± 2	0.8732	77 ± 4
I265$^{45.52}$A	6.47 ± 0.07	88 ± 3	0.0002	127 ± 4
Y341$^{6.51}$A	5.51 ± 0.13	97 ± 1	<0.0001	118 ± 6
P354^{ECL3}A	6.56 ± 0.13	109 ± 1	0.0007	169 ± 5
Y362$^{7.35}$A	6.07 ± 0.13	95 ± 0	<0.0001	137 ± 3
I366$^{7.39}$A	6.53 ± 0.12	90 ± 5	0.0004	105 ± 10
Y370$^{7.42}$A	NA	-21 ± 4	NA	122 ± 4

The response data was normalized by WT receptor within each individual experiment. Data from three independent experiments, each of which was performed in triplicate, are presented as mean ± S.E.M. NA, no activity.

Supplementary Table S6. *p*EC50 values, Emax and the expression level of mutations of MCH-binding pocket in MCHR2.

MCHR2 + MCH				
**Mutant**	***p*EC$_{50}$ ±S.E.M.**	**Emax (% WT) ± S.E.M**	***P* Value**	**Expression (%WT) ± S.E.M**
WT	8.50 ±0.08	100	> 0.9999	100
H82$^{2.53}$A	NA	NA	NA	131 ±9
L89$^{2.60}$A	8.04 ±0.02	104 ± 2	0.1322	82 ± 2
Q92$^{2.63}$A	5.04 ±0.12	25 ± 2	< 0.0001	87 ±6
W93$^{2.64}$A	5.22 ±0.06	91 ± 1	< 0.0001	78 ±4
G96^{ECL1}A	7.81 ±0.03	96 ± 0	0.0012	106 ±7
G97^{ECL1}A	NA	NA	NA	116 ±12
D113$^{3.32}$A	5.69 ± 0.09	101 ± 2	< 0.0001	85 ±2
Q117$^{3.36}$A	7.97 ± 0.06	96 ± 2	0.0352	102 ±6
F177^{ECL2}A	5.40 ± 0.07	97 ± 2	< 0.0001	97 ±4
D179^{ECL2}A	5.89 ± 0.10	85 ± 0	< 0.0001	165 ±11
V181^{ECL2}A	7.27 ± 0.07	96 ± 1	< 0.0001	93 ±1
S183^{ECL2}A	6.64 ±0. 04	95 ± 1	< 0.0001	103 ±2
C184^{ECL2}A	NA	NA	NA	115 ±7
A185$^{45.51}$W	NA	NA	NA	95 ± 6
A185$^{45.51}$F	4.90 ±0.19	96 ± 1	< 0.0001	117 ±7
F186$^{45.52}$A	7.15 ±0. 09	98 ± 0	< 0.0001	146 ±7
L188^{ECL2}A	7.26 ±0. 07	101 ± 1	< 0.0001	73 ±1
V194$^{5.35}$A	7.48 ±0.23	98 ± 1	< 0.0001	168 ±3
T198$^{5.39}$A	7.16 ± 0.07	93 ± 2	< 0.0001	108 ±4
Y267$^{6.51}$A	6.70 ±0.02	92 ± 1	< 0.0001	126 ±4
Q271$^{6.55}$A	7.56 ± 0.06	101 ± 1	< 0.0001	90 ±2
P280^{ECL3}A	6.74 ±0.09	90 ± 2	< 0.0001	122 ±7
Y285$^{7.32}$A	7.51 ±0. 24	92 ± 2	< 0.0001	165 ±5
Y288$^{7.35}$A	5.91 ± 0.03	84 ± 2	< 0.0001	96 ±5
I292$^{7.39}$A	6.89 ± 0.17	92 ± 2	< 0.0001	117 ±4
S295$^{7.42}$A	6.63 ±0.04	81 ± 2	< 0.0001	162 ± 5
Y296$^{7.43}$A	NA	NA	NA	166 ± 10

The response data was normalized by WT receptor within each individual experiment. Data from three independent experiments, each of which was performed in triplicate, are presented as mean ± S.E.M. NA, no activity.
